# A cross-sectional survey of antimicrobial stewardship in the healthcare facilities of the South West Region of Cameroon

**DOI:** 10.1017/ash.2025.10134

**Published:** 2025-09-18

**Authors:** Calvin Ncha OyongAkom, Orikomaba Obunge, Patrick A. Njukeng, Ibitein Okeafor

**Affiliations:** 1 Africa Center of Excellence in Public Health and Toxicological Research, University of Port Harcourt, Port Harcourt, Rivers State, Nigeria; 2 Global Health Systems Solutions, Douala, Cameroon; 3 Department of Medical Microbiology, University of Port Harcourt, Port Harcourt, Rivers State, Nigeria; 4 Department of Microbiology and Parasitology, University of Buea, Buea, South West Region, Cameroon; 5 Arnold School of Public Health, University of South Carolina, Columbia, SC, USA

## Abstract

**Objective:**

The objective of the study was to assess the implementation of antimicrobial stewardship (AMS) in the referral hospitals in the South West Region of Cameroon.

**Methods:**

A cross-sectional survey was carried out in November 2024 across four hospitals in the South West Region of Cameroon: Limbe Regional Hospital, Buea Regional Hospital Annex, Baptist Hospital Mutengene, and Kumba Baptist Health Center. For data collection, we used the standard WHO checklist to assess AMS activities at the healthcare facilities designed for low- and middle-income countries. Key informant interview involving the AMS focal persons of various hospitals enabled data collection.

**Results:**

Limbe Regional Hospital has the highest full implementation rate of AMS activities (67%), while Buea regional hospital is the least of fully implemented activities (19%). An average of 49% of all AMS activities are fully implemented across the healthcare facilities, partially implemented activities made up 28%, only 3% of the activities are in the planning phase but not yet started. A 13% of the activities are not implemented across the studied facilities, while 7% of the activities are not implemented but identified as a priority.

**Conclusion:**

Although the rate is still low, there is good progress in implementation of AMS activities with most activities either fully or partially implemented; however, no health facility included AMS activities in their annual plans with key performance indicators, no management of any hospital allocated financial and human resources to initiate AMS activities. Strengthening institutional commitment and AMS training are recommended.

## Introduction

Antimicrobial stewardship (AMS) refers to a wide range of procedures and actions to optimize the most efficient use of antibimicrobials.^1^ Unfortunately, Africa is still lacking the full implementation of AMS programs as many health facilities are yet to adopt the programs as was revealed in a scoping review that assessed the implementation of AMS programs in private healthcare facilities in Africa. The study showed a limited implementation of stewardship programs in the continent.^2^ A study in Nigeria found that only 35% of the hospitals had an official organizational structure and an AMS team. Only 24% have facility-specific therapy recommendations on the hospital-specific AMR patterns. Just two facilities (12%) had explicit procedures for evaluating the appropriateness of prescribed antimicrobials after 48 hours, as well as policies on approval for prescribing them. Just two sites (12%) had antimicrobial susceptibility reports for the prior year accessible, and only one hospital regularly tracked the use of antibiotics.^3^

A study in 2021 conducted worldwide found that only 10.8% of hospitals in Africa have AMS programs with reasons given as non-availability of prescription guidelines, low use of laboratory services, and a lack of laboratory capacity.^4^ Some public hospitals in South Africa have never had AMS programs as revealed in a quality study, but the hospitals with AMS programs involved practices like audits and feedback on antimicrobial use, monitoring the consumption of antimicrobials, providing regular antibiogram reports were seen in only 38.5% of public hospitals, but most hospitals (63.6%) requested blood cultures before prescriptions. The study revealed overall stewardship programs in many South African public hospitals increased correct prescriptions of antimicrobials from 10% to 80%.^4^

AMS activities identified at the facility levels were audits and thorough reviews of prescription charts, ward rounds for AMS, training, and review of prescription policies of antimicrobials.^5^

Challenges identified in South Africa were lack of specialized personnel, time, inadequate management support, poor training, lack of interest by clinicians, lack of guidelines, and financial constraints in microbiological investigations are some of the reasons for the non-effective execution of stewardship programs.^6^

Other factors that hinder effective AMS implementation across African countries include: indiscriminate antibiotic use due to self-medication and over-the-counter access without prescriptions,^7^ weak surveillance systems to track resistance patterns,^8^ and resource constraints; many healthcare facilities lack funding, trained personnel, and diagnostic tools essential for AMS programs.^8^

Despite these challenges, countries like Kenya, South Africa, and Nigeria have implemented AMS activities aligned with global objectives,^8^ some African hospitals have initiated stewardship programs focusing on improving prescribing practices and reducing unnecessary antibiotic use.^8^ A co-creation consensus approach led to the development of an AMS checklist tailored for sub-Saharan Africa. This checklist defines core AMS elements for healthcare settings in Tanzania, Zambia, Uganda, and Ghana.^9^

Antimicrobial resistance poses a significant public health challenge in Cameroon, with high rates of multidrug-resistant pathogens reported in human, animal, and environmental sectors. The 2019 systematic review by Founou et al revealed multidrug resistance rates of 45–67% among *Escherichia coli*, *Klebsiella pneumoniae*, and *Staphylococcus spp*., underscoring the need for robust AMS frameworks.^10^

Cameroon launched its National Action Plan (NAP) in 2018, adopted from the World Health Organization’s (WHO) Global Action Plan on AMR. Six objectives comprises the NAP, five of which directly align with the WHO pillars: improving awareness, strengthening surveillance, reducing infection rates, optimizing antimicrobial use, and promoting sustainable investment in combating AMR.^11,12^ Despite these efforts, implementation has faced significant challenges as the absence of a defined funding mechanism for NAP activities affects sustainability and scalability, few hospitals have adequate structures for AMR surveillance, infection prevention and control (IPC) committees, or antibiotic stewardship programs, and stakeholder coordination hinder progress. Creating public awareness about AMR remains insufficiently prioritized within the NAP objectives ^11^ and limited capacity for antimicrobial susceptibility testing hampers efforts to guide clinical decisions effectively.^13^ The lack of robust surveillance systems and limited capacity for antimicrobial susceptibility testing hinder the effective implementation of AMS.^14^

Due to the widespread of antimicrobial resistance in our clinical settings, the survey aimed to assess the effectiveness of the implementation of AMS in the Limbe Regional Hospital, Buea regional hospital annex, Baptist hospital Mutengene, Kumba Baptist Health Center in the South West Region of Cameroon to establish the current status of the program.

## Methods

### Study design

A cross-sectional survey was carried out in November 2024 where key informant interview (KII) was done involving the AMS focal persons across four hospitals. The interview was based on the indicators on the WHO checklist.

### Setting

The research was carried out in Mutengene Baptist Hospital (faith-based), Limbe Regional Hospital (public), Buea Regional Hospital Annex (public), and Kumba Baptist Health Center (faith-based). The choice of selection of the facilities was based on the fact that they are referral hospitals, except the Baptist Health Center that was added to give a balance between public and faith-based health facilities.

The implementation status of AMS activities in these referral health facilities reflect the general situation in the South West region which is one of Cameroon’s 10 regions.

Cameroon is found in the Central Africa subregion divided into 10 administrative regions and 199 health districts. The health facilities form the basis of the health system followed by the health areas, health districts, and regional and central levels in that hierarchical order.

### Methods of data collection

A standard WHO checklist to assess AMS activities at healthcare facilities designed for low- and middle-income countries was used for data collection.

The tool was adopted from Periodic National and Health-Care Facility Assessment Tools.^15^ The tool is grounded on the WHO policy on integrated AMS in human health and the WHO practical toolbox for AMS programs in healthcare facilities in low- and middle-income countries (https://apps.who.int/iris/bitstream/handle/10665/329404/9789241515481-eng.pdf). The tool was designed to assess the status of implementation of AMS programs at the national, at the sub-national, and at the health facility level. The researchers adapted the tool and removed indicators that assess AMS activities at the national and sub-national levels and concentrated on indicators at the health facilities. Thirty-six different indicators on the checklist were used to assess the hospitals’ AMS status and five standard WHO options to grade the various levels of implementation were as follows: “fully implemented,” “partially implemented,” “planned but not started,” “No,” “No but a priority,” according to the state of implementation of each AMS activity in the checklist.

The AMS focal persons of various hospitals were met and interviewed, and together with the researcher, the checklist was utilized and responses were obtained.

### Data analysis and presentation

Microsoft Office Excel was used to analyzed the data and presented in frequencies and percentages.

### Ethical considerations

The research is part of a doctoral research that is being carried out at the University of Port Harcourt; hence, an ethical clearance was gotten from the ethics committee of the University, with the reference number UPH/CEREMAD/REC/MM91/074. In Cameroon, the Baptist Convention Institutional Review Board, issued another ethical clearance, referenced; IRB202432. It enabled the researchers obtained authorization for the study in-country. A written administrative authorization was obtained from the Regional Delegate of Public Health for the South West Region, reference; P42/MINSANTE/SWR/RDPH/CB.PT/785/620. The directors and administrators of the various hospitals also issued authorizations to the researchers to access their facilities for data collection.

## Results

According to the results in Table 1, the management of only one (25%) of the healthcare facilities identified AMS as a priority, while most of the healthcare facilities of three (75%) did not consider AMS as a priority. A greater percent (75%) of health facilities team regularly monitor antibiotic susceptibility and resistance rates for a range of key indicator bacteria. Half of the facilities confirmed full implementation of regular reviews/audits of specified antibiotic therapy or clinical conditions at the healthcare facility, while 50% carried out partial implementation. Half (50%) of the studied facilities have action plans for AMS activities. No health facility (0%) fully included AMS activities in their annual plans with key performance indicators, no healthcare facility management allocated financial and human resources to initiate AMS activities but one (25%) of the facilities had an annual budget, though pointed out that it was source from an external donor for the implementation of AMS activities in the hospital. Only one facility did regular ward rounds and other interventions of AMS in some selected wards of the hospital.

**Table 1.** Showing the implementation of selected AMS activities across the hospitals

AMS Indicators	Limbe Regional Hospital	Buea Regional Hospital Annex	Baptist Hospital Mutengene	Baptist Health Center	Full Implementation *n* (%)
Is AMS identified as a priority by the healthcare facility management/leadership?	3	4	3	3	1 (25)
Is there a healthcare facility action plan in place that prioritize AMS activities?	4	3	0	4	2 (50)
Are AMS activities included in healthcare facility annual plans with key performance indicators	3	3	2	3	0 (0)
Has the healthcare facility management allocated human and financial resources to initiate AMS activities?	0	3	3	0	0 (0)
Is there a regular review/audit of specified antibiotic therapy or clinical conditions at the healthcare facility?	4	3	3	4	2 (50)
Has a budget (eg, annual) for the implementation of the healthcare facility AMS action plan been developed?	4	2	3	0	1 (25)
Does the AMS team conduct regular ward rounds and other AMS interventions in selected departments in the healthcare facility?	0	3	1	4	1 (25)
Does the AMS team regularly monitor antibiotic susceptibility and resistance rates for a range of key indicator bacteria?	4	4	1	4	3 (75)

**Key of Table 1:** 4-full implementation, 3-partial implementation, 2-planned but not started, 1-No but a priority and 0-No implementation.

In summary, nearly half (49%) of all AMS activities are fully implemented across the healthcare facilities, partially implemented activities make up 28%, and only 3% of activities are in the planning phase but not yet started. A combined 20% of activities are either not implemented (13%) or marked as “No but a priority” (7%).

Table 2 summarizes the implementation status of AMS across the four hospitals in the South West Region of Cameroon.

**Table 2.** A summary of AMS implementation across the four hospitals. The results in the table shows that Limbe Regional Hospital has the highest full implementation rate of antimicrobial stewardship activities (67%), while Buea regional hospital is the least of fully implemented activities (19%) but with a greater percentage of AMS activities (78%) partially implemented

Stewardship implementation Status	Healthcare Facilities			
	Limbe Regional Hospital *n* (%)	Buea Regional Hospital *n* (%)	Baptist Hospital Mutengene *n* (%)	Baptist Health Center Kumba *n* (%)
Fully implemented	24 (67)	7 (19)	17 (47)	22 (61)
No	8 (22)	0 (0)	4 (11)	6 (17)
No but a priority	0 (0)	0 (0)	9 (25.0)	2 (5.6)
Partially implemented	3 (8)	28 (78)	4 (111)	6 (17)
Planned but not started	1 (3)	1 (3)	2 (6)	0 (0)
Total	36 (100)	36( 100)	36 (100)	36 (100)

## Discussion

From our study, three hospitals’ AMS team regularly monitor antibiotic susceptibility and resistance rates for a range of key indicator bacteria. This is not in line to the results of a study in Nigeria where just two hospitals (12%) had antimicrobial susceptibility reports for the prior year, accessible, and only one hospital regularly tracked the use of antibiotics.^3^ In contrast to a previous study in Nigeria which showed that only 24% hospitals have facility-specific therapy recommendations on the hospital-specific AMR patterns,^3^ all of our studied facilities either carried out full implementation or partial implementation of regular review/audit of specified antibiotic therapy or clinical conditions at the healthcare facility. Our findings showed that AMS activities were not seen as a priority by the management of three out of the four hospitals studied. Similar results were seen in a study in South Africa which showed that inadequate management support and lack of interest by clinicians are some of the reasons for the non-effective execution of stewardship programs.^6^ We also found that no health facility fully included AMS activities in their annual plans with key performance indicators and no healthcare facility management allocated financial and human resources to initiate AMS activities. The findings agree with previous study in Cameroon by Amin et al which revealed the absence of a defined funding mechanism for national action plan activities affects sustainability and scalability, and few hospitals have adequate structures for AMR surveillance, or antibiotic stewardship programs, and poor stakeholder coordination hinder progress.^11^ From our research results, no full implementation of the mechanism to monitor and measure AMS activities in all the health facilities was seen which aligns with a previous assessment in Cameroon which confirmed the lack of robust surveillance systems and limited capacity for antimicrobial susceptibility testing hinder the effective implementation of AMS.^13,14^

Our results also showed that AMS committee of only one hospital did regular ward rounds, but the results of a study in South Africa showed AMS committees in most hospitals conducted activities like audits and thorough reviews of prescription charts, ward rounds for AMS, training, and review of prescription policies of antimicrobials.^5^ On the hospital-specific results, Limbe Regional Hospital has the highest full implementation rate of AMS activities, while Buea regional hospital is the least. This can be explained by the fact that the AMS was funded by an external donor as revealed by the focal person. Although sustained partnership is important in AMS activities implementation, local budget for the activities will guarantee sustainability.

## Conclusion

A summary of the findings showed that nearly half of the studied facilities fully implemented AMS activities. This suggests that while there is good progress in implementation of AMS activities with most of the activities either fully or partially implemented, there is still room for improvement, particularly in addressing the few critical activities that have not been implemented yet. The lack of allocation of financial resources for AMS activities across the hospitals and the non-inclusion of AMS activities in annual plans with key performance indicators are set back to effective implementation of its activities.

### Recommendations

- The management of hospitals should actively show full support to AMS activities to ensure successful implementation.
- AMS should be prioritized and included in the annual plans of all health facilities with measurable key performance indicators.
- The management of hospitals should allocate annual budgets for the full implementation of AMS activities and partner with external donors to complement the funding.
- A focal person should be designated under the supervision of the AMS focal person, to monitor and measure AMS activities in all the health facilities.
- AMS committee members of various hospitals should undergo regular trainings on AMS.
- Ward rounds should be regularly done by the AMS committee members to identify compliance with antibiotics use.
- Weekly meetings should be organized by the AMS committee members to inform clinicians and other team members the surveillance reports of antimicrobials used in the hospital vis-à-vis susceptibility reports.
- AMS committees should regularly provide hospital-based specific recommendations on the antimicrobial resistance rate of the hospitals to guide prescriptions.
